# The Population Effective Dose of Medical Computed Tomography Examinations in Taiwan for 2013

**DOI:** 10.1371/journal.pone.0165526

**Published:** 2016-10-27

**Authors:** Da-Ming Yeh, Hui-Yu Tsai, Yen-Sheng Tyan, Yu-Cheng Chang, Lung-Kwang Pan, Tou-Rong Chen

**Affiliations:** 1 Department of Medical Image, Chung Shan Medical University Hospital, Taichung, Taiwan; 2 Department of Medical Imaging and Radiological Sciences, Chung Shan Medical University, Taichung, Taiwan; 3 Department of Medical Imaging and Radiological Sciences, Central Taiwan University of Science and Technology, Taichung, Taiwan; 4 Department of Medical Imaging and Radiological Sciences, College of Medicine, Chang Gung University, Taoyuan, Taiwan; 5 Medical Physics Research Center, Institute for Radiological Research, Chang Gung University / Chang Gung Memorial Hospital, Taoyuan, Taiwan; 6 Department of Medical Imaging and Intervention, Chang Gung Memorial Hospital Linkou, College of Medicine, Chang Gung University, Taoyuan, Taiwan; 7 School of Medicine, Chung Shan Medical University, Taichung, Taiwan; Mizoram University, INDIA

## Abstract

**Purpose:**

To evaluate the annual effective dose per capita attributed to computed tomography (CT) examinations in 2013 and to predict the population effective dose from 2000 to 2013 in Taiwan.

**Methods:**

A CT examination database collected from 30 hospitals was divided into 22 procedures and classified into six regions: head, neck, chest, abdomen, pelvis, and other, respectively. The effective doses in different regions were evaluated by dose-length product (DLP) multiplied by conversion factors.

**Results:**

The CT scan dose parameters were collected from 4,407 patients. For the six scanned regions, the percentages of patients scanned were: head (39.8%), neck (3.9%), chest (23.3%), abdomen (26.7%), pelvis (4.8%), and other (1.6%), respectively. The DLPs per patient (mGy·cm/patient) were head (1,071±225), neck (1,103±615), chest (724±509), abdomen (1,315±550), pelvis (1,231±620) and other (1,407±937), respectively. The number of CT examinations increased rapidly, with an average annual growth rate of 7.6%. The number of CT examinations in 2013 was 2.6 times that in 2000. The population effective dose was 0.30 mSv per capita in 2000 and increased to 0.74 mSv per capita in 2013, with an annual growth rate of 7.2%. The growth trend indicates that the effective dose will continue to rise in Taiwan.

**Conclusion:**

Some strategies should be applied to cope with this growth. Defining the CT dose reference level stipulated in official recommendations and encouraging the use of iterative reconstruction imaging instead of filtered back-projection imaging could be a useful method for optimizing the effective dose and image quality.

## Introduction

The average effective dose per capita in the U.S. population was 6.2 mSv for 2006. The dose was a combination of natural background radiation (50%), medical exposure of patients (48%), and consumer products (2%) [1]. The largest contributor to the population dose from medical exposure is computed tomography (CT) examination. The population effective dose of CT examination is 1.47 mSv, and this dose accounts for 49% of the medical exposure in the U.S. From 1980 to 2006, the effective dose due to CT examination increased 92-fold [1]. A high effective dose potentially means a higher risk of cancer. Berrington de González [2] estimated in 2007 that approximately 29,000 (95% uncertainty limits, 15,000–45,000) future cancers could be related to CT scans performed in the U.S.

The annual effective dose attributed to natural background radiation was about 1.62 mSv [3], and to medical exposures, about 0.74 mSv in Taiwan for 2008 [4]. In 2008, the frequency of CT examinations accounted for only 4.7% of total medical exposures, but the corresponding contribution to the effective dose was as high as 50.8% [4]. However, the study assumed a CT examination on a patient consisting of a single CT scan, where a scan refers to a single sequence of rotations [4]. Because some CT procedures consist of two or more scans and patients may undergo multiple repeat procedures, estimating the collective effective dose requires estimating the number of scans associated with these procedures.

The National Health Insurance (NHI) program in Taiwan, launched in 1995, is a compulsory insurance program for all residents of Taiwan promoted. Soon after its implementation, the NHI extended its coverage to 59%, and that figure rose to 100% by 2013. The NHI covers virtually the entire population of Taiwan. The Bureau of NHI is part of the Ministry of Health and Welfare (MOHW). This study used information on the total number of patients who underwent CT examinations released by the Department of Statistics of the MOHW. However, the registrations of MOHW data or the NHI program only classified the CT procedures into two broad categories: head scans and body scans. Such a classification does not meet the requirements for effective dose estimates based on CT scans of specific body parts: the head, neck, chest, abdomen, and pelvis. Dose parameters, namely the CT dose index (CTDI) and scan length, or dose-length product (DLP), are not available in the MOHW database. In addition, the data do not specify the frequency of scans per procedure or per patient.

This study was conducted to establish a Taiwan CT Scan Database (TCTD) containing scan parameter details. The goal was to analyze the DLPs per patient of different scanned regions from the database to determine the annual effective dose per capita in Taiwan for 2000 to 2013, after multiplying the DLP with the conversion factor (*k* value), and with the total number of patients from the MOHW.

## Materials and Methods

### CT scan database

Radiology technologists at 30 clinical CT units in 30 hospitals in Taiwan recorded the actual CT scan dose parameters on a form, which included the scanned regions, frequency of scans, scan protocol, DLP per scan, and DLP per patient. If there was no DLP information on the screen of the CT unit, both the CTDI and scan length were recorded by the technologist. The technologists did not record the weight, high, age, name, medical record number of hospital, and any patient’s personal information. The 30 hospitals, which were evenly distributed throughout Taiwan, were 5 medical centers, 14 metropolitan hospitals, and 11 local community hospitals. The period of data collection was one to two weeks for each CT unit, and the database of the study was collected from July 2013 to August 2014.

### Conversion factor

The conversion factor, sometimes called the *k* value, is a factor for converting DLP to effective dose. The effective dose can be estimated by multiplying the scanned DLP by the corresponding *k* value. The effective dose is related to the scan region, which directly corresponds to different organs with different tissue weighting factors [5, 6]. The conversion factors of the American Association of Physicists in Medicine (AAPM) Report No. 96 and the European (EUR) published EUR16262 report were often used in the past [7, 8]. However, the tissue weighting factors have been updated in a new version of the ICRP, ICRP report No. 103 [6]. The k values based on ICRP No. 103 recommended by Deak [9] were adopted in our study. The region of other in the study was divided into two parts, extremities and remaining regions. For the extremity region, we adopted the *k* value of the knee, 0.0004 mSv·mGy^-1^·cm^-1^, recommended by Saltybaeva [10]. For the remaining CT examinations, we adopted the *k* value of 0.010 mSv·mGy^-1^·cm^-1^ from the mean value of five *k* values in different regions recommended by Deak [9].

### Scanned regions

CT procedures were grouped by body segment or organ examination for convenience of analysis. The six regions were the head, neck, chest, abdomen, pelvis, and other regions, respectively. The CT examinations in the database were collected from 30 hospitals and divided into 22 procedures, which were then classified into six regions. No exclusion criteria were applied to the collected sample. When the scan length covered two or more regions, a weighting factor was assigned to each region. The classification and weighting factors are shown in Table 1.

**Table 1:** Weighting factors of the 22 procedures classified into six regions.

Procedure	Head	Neck	Chest	Abdomen	Pelvis	Other
Brain	1.00	-	-	-	-	-
Sinus	1.00	-	-	-	-	-
Facial bone	1.00	-	-	-	-	-
Neck	-	1.00	-	-	-	-
C spine	-	1.00	-	-	-	-
Nasopharynx	-	1.00	-	-	-	-
Head to neck	0.50	0.50	-	-	-	-
Routine chest	-	-	1.00	-	-	-
Chest low-dose	-	-	1.00	-	-	-
Chest high-resolution	-	-	1.00	-	-	-
Chest to abdomen	-	-	1.00	-	-	-
Cardiac scan	-	-	1.00	-	-	-
Routine abdomen	-	-	-	1.00	-	-
Liver	-	-	-	1.00	-	-
L spine	-	-	-	1.00	-	-
Urography (CTU)	-	-	-	1.00	-	-
Abdomen to pelvis	-	-	-	0.50	0.50	-
Chest to pelvis	-	-	0.33	0.33	0.33	-
Whole body	0.13	0.13	0.25	0.25	0.25	-
Pelvis	-	-	-	-	1.00	-
Extremities	-	-	-	-	-	1.00
Remainder	-	-	-	-	-	1.00

## Results

### Patient distribution of database

The patients were 2,479 males and 1,928 females, for a total of 4,407 patients. The number of patients and percentages corresponding to the 22 procedures are listed in Table 2.

**Table 2:** Patient number and percentage corresponding to 22 procedures.

Procedure	Patient no.	%
Brain	1624	36.8
Sinus	79	1.8
Facial bone	28	0.6
Neck	113	2.6
C spine	30	0.7
Nasopharynx	6	0.1
Head to neck	41	0.9
Routine chest	582	13.2
Chest low-dose	221	5
Chest high-resolution	73	1.7
Chest to abdomen	32	0.7
Cardiac	94	2.1
Routine abdomen	510	11.6
Liver	368	8.4
L spine	70	1.6
CTU	39	0.9
Abdomen to pelvis	330	7.5
Chest to pelvis	62	1.4
Whole body	14	0.3
Pelvis	22	0.5
Extremities	50	1.1
Remainder	19	0.4
Total	4407	100

The table shows the percentages of patients receiving scans for each region in descending order: brain (36.8%), routine chest (13.2%), routine abdomen (11.6%), liver (8.4%), abdomen and pelvis (7.5%), chest low-dose (5.0%). The above six procedures (≥5.0%) accounted for 82.5% of the procedures.

Table 3 shows number of patients, and percentages of the six regions, according to the rules of Table 1. The number of patients and percentages were as follows: head, abdomen, chest, pelvis, neck, other. Head examinations had the highest percentage, 39.8%, of the total number of patients, followed by the abdomen (26.7%) and chest (23.3%). The pelvis, neck, and other amounted to 8.7%. Brain examinations of the head region were the largest proportion, 36.8%, of the patients for all regions. For the chest region, routine chest procedures comprised 13.2%, low-dose chest procedures comprised 5.0%, and high-resolution chest procedures comprised 1.7%. Cardiac CT procedures comprised 2.1% of scans in the chest region.

**Table 3:** Patient numbers and percentages in the six regions.

Region	Patient no.	%
Head	1754	39.8
Neck	171	3.9
Chest	1026	23.3
Abdomen	1176	26.7
Pelvis	211	4.8
Other	69	1.6
Total	4407	100

The abdomen ranked second in the total number of patients. Routine abdomen procedures accounted for 11.6%, and liver CT procedures accounted for 8.4%. The high number of patients receiving liver CT could be related to Taiwan's high prevalence of liver disease. The prevalence of liver cancer for males and females in Taiwan has been reported as 26 and 8 people per 10 million [11, 12]. In addition, the abdomen to pelvis scan, one of the cross-region procedures, accounted for 7.5%. This percentage is much higher than those of other cross-region procedures: 0.7% for chest to abdomen, 1.4% for chest to pelvis, and 0.3% for whole body procedures. The other region contributed 1.6% of the total number of patients, with extremity procedures accounting for the largest part, 1.1%.

### DLP distribution and frequency of scan per patient

The DLP per scan, frequency of scan per patient, and DLP per patient are presented in Table 4. The ranking of DLPs of 22 procedures for each scan was as follows: Whole body, Chest to Pelvis, Extremities, Head to neck, Brain. Roughly, this indicates that the longer the scan length, the greater the DLP value. The frequencies of scans per patient (scan/patient) were in the order of Liver (3.8), CTU (3.5), Cardiac (2.3), Chest to Abdomen (2.2), Neck (1.9), C spine (1.9). Liver CT examinations could include the un-enhanced phase, pre-contrast, arterial phase, portal venous phase, and delayed phase. These sets of liver scans, known as 3-phase, 4-phase, or multiphase CT scans, can sometimes help to tell a benign tumor from a malignant one and have the largest frequency of scans per patient. Second was the CTU procedure, with a mean frequency of 3.5 scans per patient. The head CT procedure, the procedure performed the most in Taiwan, had a mean frequency of 1.2 scans per patient.

**Table 4:** DLP per scan, frequency of scan per patient, and DLP per patient (mGy·cm/patient).

Procedure	DLP/scan		scan/procedure	DLP/patient	
	Mean	std	Mean	Mean	std
Brain	930	239	1.18	1093	281
Sinus	491	226	1.12	551	254
Facial bone	455	294	1.60	726	469
Neck	588	349	1.90	1119	664
C spine	330	182	1.90	629	347
Nasopharynx	613	441	1.33	818	589
Head to neck	975	407	1.75	1708	712
Routine chest	404	189	1.71	689	322
Chest low-dose	177	133	1.17	206	155
Chest high-resolution	492	329	1.01	497	332
Chest to abdomen	665	258	2.21	1469	570
Cardiac	818	597	2.30	1877	1371
Routine abdomen	541	252	1.84	996	464
Liver	483	192	3.78	1827	728
L spine	552	286	1.16	638	331
CTU	610	328	3.53	2156	1160
Abdomen to pelvis	738	265	1.67	1230	442
Chest to pelvis	1049	415	1.32	1388	549
Whole body	1552	331	1.27	1974	421
Pelvis	691	496	1.46	1012	726
Extremities	1023	819	1.60	1635	1310
Remainder	464	414	1.75	811	724
Mean	642	212	1.67	1070	354

The DLP for a single patient can be calculated by multiplying the DLP per scan by the scan frequency per patient. The rightmost column of Table 4 shows the DLPs per patient larger than 1500 mGy·cm: CTU, Whole body, Cardiac, Liver, Head to neck, Extremities. The procedures of Chest low-dose (206 mGy·cm) and Chest high-resolution (497 mGy·cm) had DLPs per patient of less than 500 mGy·cm. The 22 procedures and corresponding numbers of patients that were classified and transferred to six regions of CT examinations according to the attributed rules shown in Table 1 are presented in Table 5.

**Table 5:** Means and standard deviations for DLP per scan (mGy·cm/scan), scan frequency per patient (scans/patient), and DLP per patient (mGy·cm/patient) on the six regions of CT examinations.

Region	DLP/scan		scan/procedure	DLP/patient	
	Mean	std	Mean	Mean	std
Head	902	190	1.19	1071	225
Neck	591	330	1.87	1103	615
Chest	452	318	1.60	724	509
Abdomen	542	226	2.43	1315	550
Pelvis	770	388	1.60	1231	620
Other	858	572	1.64	1407	937
Total	641	212	1.67	1070	354

The head region had the largest mean value and smallest standard deviation of DLP per scan. The other region had a large fluctuation from its standard deviation. The frequency of scans (scans/patients) was calculated on the basis of the 22 procedures in Table 4 and the weighting of the corresponding numbers of patients.

## Analysis and Discussion

### CT operation information of 2000 to 2013 in Taiwan

To calculate the annual population effective dose per capita, the study obtained the total population number from the Ministry of the Interior [13] and the number of patients receiving CT examinations from the MOHW [14]. The population size, number of patients receiving CT examinations, patient frequency per 1,000 people, CT units [15], and patient load (patient examinations per unit in a week) from 2000 to 2013 in Taiwan are shown in Table 6. The compound annual growth rate (CAGR) was used to calculate these CT related indicators in the table.

**Table 6:** The number of population, number of patients receiving CT examination, patient frequency per 1000 people (patient/1000 person), CT units, patient load, and the CAGR of 2000 to 2013 in Taiwan.

Year	Population	Patient	Frequency	CT units	Patient load
2000	22,276,672	689,837	31	308	45
2001	22,405,568	707,235	32	310	46
2002	22,520,776	806,864	36	317	51
2003	22,604,550	849,518	38	325	52
2004	22,689,122	978,265	43	318	62
2005	22,770,383	981,805	43	321	61
2006	22,876,527	1,029,200	45	320	64
2007	22,958,360	1,141,864	50	318	72
2008	23,037,031	1,268,921	55	321	79
2009	23,119,772	1,391,606	60	331	84
2010	23,162,123	1,466,001	63	329	89
2011	23,224,912	1,568,422	68	338	93
2012	23,315,822	1,697,043	73	343	99
2013	23,379,129	1,794872	77	345	104
CAGR	0.4%	7.6%	7.2%	0.9%	6.7%

From 2000 to 2013, the size of Taiwan's population remained stable, growing only 5% over 13 years, with an average annual growth of 0.4% CAGR [13]. However, the number of patients receiving CT examinations increased rapidly, with an average annual growth of 7.6%. The number of CT examinations in 2013 was 2.6 times that in 2000 [14]. In 2013, the frequency of CT examinations in Taiwan was 77 per 1,000 people, while in 2006; it was only 45 per 1,000 people. In 2006, the CT examination rate in the US was 207 per 1,000 people, and in the UK, it was about 50 per 1,000 people [16]. The total number of clinical CT units was 308 in 2000, and that number grew to 345 units in 2013, with an average annual growth rate of 0.9% [15]. The patient loads are listed in the rightmost column of Table 6. The average number of patients per CT unit in a week was 45 in 2000, and that number increased to 104 in 2013, with a CAGR of 6.7%.

### Effective dose of six CT scan regions

The effective dose for a single scan and a patient can be calculated by multiplying the DLP per scan (mGy·cm/scan) and DLP per patient (mGy·cm/patient) in Table 5 by the *k* values recommended by Deak [9], as detailed in Table 7.

**Table 7:** The effective dose per scan and per patient calculated by the DLP survey data and *k* values.

Region	Deak	mSv/scan		mSv/patient	
	k value	Mean	std	Mean	std
Head	0.0019	1.7	0.4	2.0	0.4
Neck	0.0052	3.1	1.7	5.7	3.2
Chest	0.0146	6.6	4.6	10.6	7.4
Abdomen	0.0153	8.3	3.5	20.1	8.4
Pelvis	0.0129	9.9	5.0	15.9	8.0
Other	0.0100	8.6	5.7	4.3	9.4
Total	-	5.8	1.9	9.7	3.2

The average effective doses for the six regions for single CT scans were, in decreasing order, the pelvis (9.9 mSv), abdomen (8.3 mSv), chest (6.6 mSv), neck (3.1 mSv), other (2.6 mSv), head (1.7 mSv). Overall, the effective dose for a single scan is about 5.8 mSv.

Because the scan frequencies per patient (scan/patient) in the six regions of CT examination were different, as shown in Table 5, the order of the effective doses per patient was also rearranged. Since the abdomen region had the highest scan frequency, 2.43 scans per patient, the maximum effective dose per patient occurred in the abdomen region. The average effective doses per patient were, in the descending order: abdomen (20.1 mSv), pelvis (15.9 mSv), chest (10.6 mSv), neck (5.7 mSv), other (4.3 mSv), head (2.0 mSv).

### Population effective dose per capita

In Taiwan, as of 2013, the number of patients who had received CT examinations was 1,794,872 [14]. It is assumed that the behavior of CT examinations in Taiwan matches the results from analysis of the survey data. The numbers of patients for the six CT scan regions were calculated by applying the results shown in Table 3 to the total number of patients in Taiwan. The collective effective dose was obtained by multiplying the effective dose per patient in Table 7 by the number of patients. Dividing by the population, 23,379,129 people [13], we calculated the average population effective dose (mSv per capita) for 2013 shown in Table 8.

**Table 8:** Expected patient numbers, effective dose per patient, collective effective dose, effective dose per capita, and their contributions on six scanned regions.

Region	Expected patient	mSv/patient	Collective ED (Sv)	ED/capita (mSv)	Contribution
Head	714365	2.0	1454	0.06	8.4%
Neck	69645	5.7	399	0.02	2.3%
Chest	417866	10.6	4415	0.19	25.4%
Abdomen	478958	20.1	9635	0.41	55.4%
Pelvis	85936	15.9	1364	0.06	7.8%
Other	28102	4.3	120	0.01	0.7%
Total	1794872	9.7	17387	0.74	100.0%

The population effective dose of Taiwan was 0.74 mSv per capita in 2013. CT scans of the abdomen contributed the largest proportion, 55.4%, of the effective dose. Scans of the chest region ranked second, at 25.4%. Together, abdomen and chest examinations accounted for 80.8% of the overall population effective dose. Although the proportion of scans of the head region, 39.8%, was 8.3 times that of the scanned proportion of the pelvis region, 4.8%, the contribution to the effective dose from head examinations, 8.4%, was only slightly higher than that from pelvis examinations, 7.8%.

**Fig 1:**
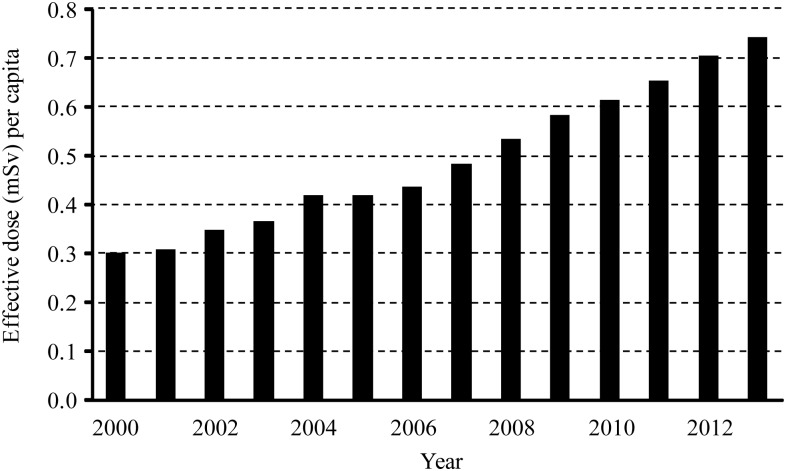
Annual growth of population effective dose in Taiwan from 2000 to 2013.

Furthermore, it was assumed that the proportions of CT examinations from 2000 to 2013 were similar to those of the survey database collected in the study. Then after application of the population and number of patients in Table 6, the population effective dose was evaluated. The population effective dose was 0.30 mSv per capita in 2000, and that number increased to 0.74 mSv per capita in 2013, with an annual growth rate (CAGR) of 7.2%. Fig 1 presents the annual population effective doses from 2000 to 2013.

To compare our results with those of Chen [4], we calculated the population effective dose for 2008 with a total number of 1,268,921 patients. The result was 0.53 mSv per capita, which is 1.4 times the 0.38-mSv per capita reported by Chen. This difference can be explained by differences in data; the number unit of CT examinations released by the MOHW is patients, rather than scan frequencies.

The population effective dose of CT examinations in 2013 was 2.5 times that in 2000, and the growth trend forecast in Fig 1 suggests that the effective dose will continue to rise in Taiwan. To reduce this growth, effective methods should be used to both optimize the frequency of CT scans and reduce the DLP per scan, particularly those in the abdomen and chest regions. Reduction in DLP may be achieved by optimizing scanning protocols to eliminate over-scanning. Regarding the methods for optimizing the effective dose, in addition to carefully reducing the length of redundant scans, other image reconstruction methods can be used [17]. Studies have shown that iterative reconstruction (IR) imaging can produce images with less noise and a higher contrast-to-noise ratio (CNR) than those produced through filtered back-projection (FBP). Because doctors are familiar with FBP images, IR images are rarely used in clinical settings in Taiwan. Through the training education of radiological technicians and doctors, promoting the use of IR imaging instead of FBP imaging may be a viable method for optimizing the effective dose and image quality. Defining CT reference levels of DLP or CTDI_v_ may also be a suitable approach to lowering or optimizing the collective effective dose [17, 18]. Currently, no official dose reference level has been suggested, and further research could be conducted to analyze the reference level in Taiwan.

### Effective dose contributions of 22 CT examinations

One aim of this study was to try to determine the contributions to the effective dose of 22 CT examination procedures in Taiwan. The effective doses per scan and per patient were calculated by multiplying the DLPs of 22 procedures in Table 4 by the corresponding *k* value of each region, as presented in Table 9. In addition, we used the same steps as used in producing Table 8 to calculate the contributions of the collective effective dose, but replaced the 6 regions with the 22 procedures.

**Table 9:** Effective dose (mSv) per scan, per patient, and contribution percentage of collective effective dose for 22 procedures in Taiwan.

Procedure	mSv/scan		mSv/patient		Contribution
	Mean	std	Mean	std	%
Brain	1.8	0.5	2.1	4.5	7.9
Sinus	0.9	0.4	1.0	0.1	0.2
Facial bone	0.9	0.6	1.4	0.1	0.1
Neck	3.1	1.8	5.8	1.2	1.5
C spine	1.7	0.9	3.3	0.1	0.2
Nasopharynx	3.2	2.3	4.2	0.1	0.1
Head to neck	3.4	1.4	6.0	0.4	0.6
Routine chest	5.9	2.8	10.1	8.8	13.8
Chest low-dose	2.6	1.9	3.0	1.0	1.6
Chest high-resolution	7.2	4.8	7.3	0.8	1.2
Chest to abdomen	9.7	3.8	21.5	1.1	1.6
Cardiac	11.9	8.7	27.4	3.3	6.1
Routine abdomen	8.3	3.9	15.2	9.5	18.2
Liver	7.4	2.9	28.0	13.4	24.2
L spine	8.4	4.4	9.8	0.9	1.6
CTU	9.3	5.0	32.9	1.6	3.0
Abdomen to pelvis	10.4	3.7	17.3	7.8	13.4
Chest to pelvis	14.9	5.9	19.7	2.1	2.9
Whole body	17.9	3.8	22.7	0.3	0.7
Pelvis	8.8	6.3	12.8	0.5	0.7
Extremities	0.4	0.3	0.7	0.0	0.1
Remaider	4.6	4.1	8.1	0.3	0.4
Mean	5.8	1.9	9.7	57.6	100.0

Among the 22 procedures, the effective doses for single scans of the top five procedures were, in descending order, as follows: Whole body (17.9 mSv), Chest to pelvis (14.9 mSv), Cardiac (11.9 mSv), Abdomen to pelvis (10.4 mSv), Chest to abdomen (9.7 mSv).

The scan frequencies of the 22 procedures were different. After multiplication by the scan frequency (scan/patient), the top five procedures of effective dose for a patient were, in descending order, as follows: CTU (32.9 mSv), Liver (28.0 mSv), Cardiac (27.4 mSv), Whole body (22.7 mSv), Chest to abdomen (21.5 mSv). The CTU examination had the largest effective dose per patient, mainly due to the effective dose per scan, 9.3 mSv/scan, and high scan frequency per patient, 3.5 scans/patient. Liver CT, which had the highest frequency of 3.7 times, was the second largest effective dose. Both CTU and Liver CT examinations are focused on the abdomen. On the other hand, the procedures with lower effective doses were those focused on organs with lower tissue weighting factors. For the Extremities procedure, only a small portion of the critical organs and tissues was considered. For the Brain procedure, the tissue weighting factor of the brain is only 0.01. It follows that the Brain procedure contributed less to the effective dose per scan or per patient.

The areas scanned, ranked by percentage of patients, were, in descending order, as follows: Brain (36.8%), Routine chest (13.2%), Routine abdomen (11.6%), Liver (8.4%), Abdomen to pelvis (7.5%), Chest low-dose (5.0%), Neck (2.6%), Cardiac (2.1%); all are higher than 2% in Table 2. However, the contributions to the collective effective dose were Liver (24.2%), Routine abdomen (18.2%), Routine chest (13.8%), Abdomen to pelvis (13.4%), Brain (7.9%), Cardiac (6.1%), CTU (3.0%), Chest to pelvis (2.9%). The collective effective dose was mainly determined by the number of patients and the effective dose for different procedures. Although the Brain procedure had the largest number of patients, its collective effective dose was ranked only fifth. On the other hand, the percentage of patients, 8.4%, who received Liver CT was less than a quarter of those receiving the Brain procedure. The contribution of the collective dose of the Liver CT procedure was 24.2% due to its high effective dose per patient, 28.0 mSv. Of the procedures that contributed more than 2.0% to the collective effective dose, most were located in the abdomen region, followed by the chest region and then the head region. This result is consistent with the contents of Table 8, in that the primary contribution of the effective dose is the scan in the abdominal region, and the secondary contribution is in the chest region.

### Limitations

The collected DLPs include children’s DLPs, but we applied only one adult conversion factor to all of the patients. Because the effective dose conversion factors for children are larger than those for adults, this could cause the actual population effective dose to be higher than the assessed value (0.74 mSv per captia). Only 30 units of CT were survey in the study, which is 8.7% of the total CT number (345 units). Mapping only 8.7% of the data to represent the scanned data of the whole of Taiwan could be a limitation.

Over the years, single-slice CT (SSCT) was gradually replaced by the multislice CT (MSCT). Brix indicated that four-slice CT presents higher DLP values compared with SSCT [19]. Karim indicated that the DLP values for MSCT were slightly higher than those for SSCT [17]. Currently, MSCT is performed for various slices, ranging from a single slice to 640 slices. This means that the slice distribution of MSCT for different slices is changing annually. Mapping the DLP for 2013 to the DLP for other years could be problematic.

## Conclusion

The population effective dose of CT examinations in 2013 was 2.5 times that in 2000, and the growth trend suggests that it will continue to rise in Taiwan. CT abdominal scans accounted for more than half the dose contribution, particularly liver scans. To study and reduce the incidence of liver cancer is a critical and ongoing concern in Taiwan. Defining the CT dose reference level promoted by official recommendations and encouraging the use of iterative reconstruction imaging instead of filtered back-projection imaging could be useful strategies for coping with the effective dose growth.

## Supporting Information

S1 File CT survey DLP data. (XLSX)
